# Lockdown Due to COVID-19 and Its Consequences on Diet, Physical Activity, Lifestyle, and Other Aspects of Daily Life Worldwide: A Narrative Review

**DOI:** 10.3390/ijerph19116832

**Published:** 2022-06-02

**Authors:** Teresa Rubio-Tomás, Maria Skouroliakou, Dimitrios Ntountaniotis

**Affiliations:** 1 School of Medicine, University of Crete, 70013 Herakleion, Greece; teresa.rubio.t111@gmail.com; 2 Department of Nutrition and Dietetics, Harokopio University of Athens, 17671 Athens, Greece; mskour@hua.gr; 3 Laboratory of Organic Chemistry, Chemistry Department, National and Kapodistrian University of Athens, 11527 Athens, Greece

**Keywords:** COVID-19, pandemics, lockdown, diet, physical activity, lifestyle

## Abstract

The novel coronavirus, termed severe acute respiratory syndrome coronavirus 2 (SARS-CoV-2), is responsible for the disease called coronavirus disease 2019 (COVID-19). Besides the important rates of mortality and morbidity directly attributed to the infection itself, many studies detected an important shift towards mostly unhealthy lifestyle patterns in previously healthy non-infected populations all around the world. Although most of the changes in lifestyle had or will have a negative impact on general population health status, some findings are encouraging. Notwithstanding that there was an obvious necessity for governments to apply national lockdowns, it is also necessary to identify and comprehend the consequences they have caused. A narrative literature review was performed, based on scientific articles and previous reviews. An accurate description of changes in eating habits and alcohol consumption, physical activity, mental health, daily routines, economic impacts, and broader effects on society is provided for each continent and different age groups through this review. The volume of selected scientific surveys encompasses approximately 400,000 persons.

## 1. Introduction

The novel coronavirus, termed severe acute respiratory syndrome coronavirus 2 (SARS-CoV-2), is responsible for the disease called coronavirus disease 2019 (COVID-19). It originated in Wuhan, China, where a cluster of pneumonia patients of unknown etiology was described on 31 December 2019 and subsequently attributed to SARS-CoV-2 on 9 January 2020. By February 2020 the virus had spread to many countries [1,2] and on 11 March 2020 it was declared a pandemic by the World Health Organization (WHO) [3].

Between 31 December 2019 and the twentieth week of 2022, the official worldwide number of COVID-19 cases was 524,468,542 and COVID-19-related deaths totaled 6,291,947 [4].

Following the recommendations released by the WHO, each government implemented a wide range of mitigation strategies against the COVID-19 pandemic, ranging from school and university closures, park closures, restaurant and shop closures or reductions in capacity, to home confinement, curfews, population control by means of technological devices, restriction of mobility inside the country, and border closures, as well as the creation of new hospitals and the transformation of facilities such as hotels or congress buildings into appropriate healthcare settings [5,6,7]. Although an international online survey performed at the very beginning of the pandemic already envisaged detrimental effects of mobility restrictions on eating behavior and physical activity, the consequences of COVID-19 on each geographical area differ widely [8]. Furthermore, scientific research aimed at finding effective treatments and vaccines for the virus has been intense, leading to the recent onset of the vaccination campaign in many countries [9,10].

In this review, we will focus on the consequences to the general population, mainly regarding eating habits, physical activity, lifestyle, and psychology, because of the restrictions applied during lockdown. Some additional consequences related to essential aspects of human society and the economy are reported. The data are organized per continent and the consequences to specific subgroups are considered (in each continent). The profound understanding of all the problems that arise should lead to better solutions.

Before presenting these data, a detailed list of the effects due to lockdown is provided in the Table 1.

## 2. Materials and Methods

In order to write the current narrative literature review, the co-authors accessed Scopus and PubMed. The key phrases that were used during search were the following:

- COVID-19 (or pandemic) and lifestyle
- COVID-19 (or pandemic) and nutrition
- COVID-19 (or pandemic) and dietary patterns
- COVID-19 (or pandemic) and nutritional trend
- COVID-19 (or pandemic) and nutritional impact
- COVID-19 (or pandemic) and future food consumption
- COVID-19 (or pandemic) and potential nutrition
- COVID-19 (or pandemic) and implications for nutrition
- COVID-19 (or pandemic) and nutrients
- COVID-19 (or pandemic) and diet
- COVID-19 (or pandemic) and aging
- COVID-19 (or pandemic) and physical activity
- COVID-19 (or pandemic) and exercise
- COVID-19 (or pandemic) and habits
- Lockdown and diet
- Lockdown and lifestyle
- Lockdown and nutrition
- Lockdown and dietary patterns
- Lockdown and physical activity
- Lockdown and exercise
- Lockdown and habits

**Figure 1:**
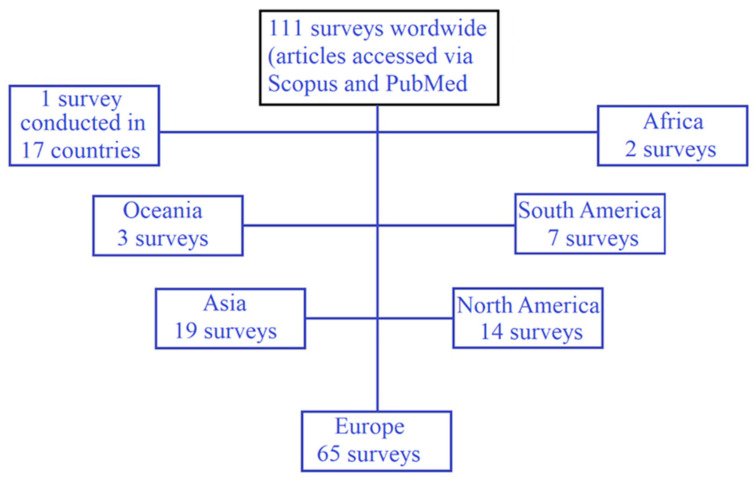
Distribution of surveys by continent (referring to COVID-19 consequences).

After completing the search and performing a check for duplicate entries in the final choice of articles, the aim was to group the articles. Grouping was performed firstly based on surveys by continent and then articles per continent were separated by further subgrouping referring to the consequences of COVID-19 by age. Two inclusion criteria were under consideration: (i) the most recent articles were selected in order to understand the effects derived from the lockdown (time is necessary in order to estimate the consequences); and (ii) an important sample size (N) was necessary. (More than 300 respondents per survey participated in 86.36% of the articles. In addition, more than 1000 respondents participated in 65.45%). Only 15 articles with fewer than 300 participants were included, either because interesting findings were reported, or because a greater variety of nationalities was preferred. (Regarding some countries, only one article was found during the search). The distribution of the surveys by continent is illustrated in Figure 1.

**Figure 2:**
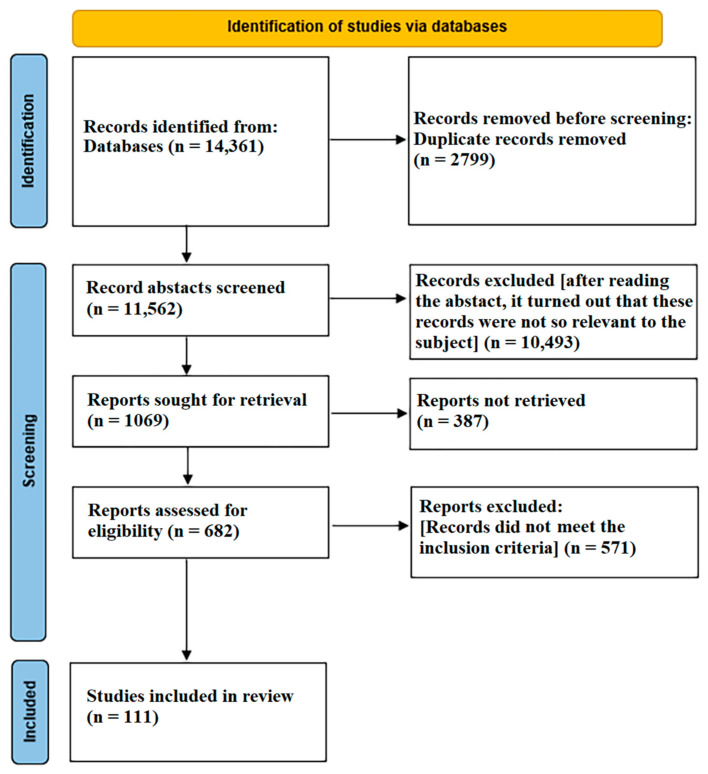
PRISMA flow diagram for the narrative review which included searches of databases (March 2020–December 2021).

A PRISMA flow diagram for this review is presented in Figure 2.

The impact/changes observed during the COVID-19 pandemic (in Asia, Europe, North America, South America, Oceania and Africa) are presented separately in the following sections. There is a table per continent, which summarizes the references cited in the text. In addition, similar trends during the pandemic (concerning parameters in which the same effects are observed in different countries) were recorded in this review.

## 3. Results

### 3.1. Asia

To date, Asia has reported 132,563,392 coronavirus cases [4]. China was the first country to face the pandemic. The Chinese citizens found themselves in unprecedented situations that they did not know how to deal with. China imposed a lockdown in Wuhan and other cities in Hubei during January 2020 (affecting millions of people) and then other virus-control measures were implemented in other regions [67,68]. More lockdowns were applied to other nations during the pandemic. The consequences are discussed below. Regardless of other national measures, many governments tried to grapple with critical agricultural issues through the implementation of key policies [66]. A brief summary of studies related to the consequences of the lockdown in Asian populations is presented in Table 2.

#### 3.1.1. Children and Adolescents

An online survey in China aimed to determine the levels of health-related behaviors (physical activity, screen exposure, and sleep status) among students from primary, secondary, and high schools during the pandemic. A total of 10,416 students participated. The study revealed that the school closure resulted in negative effects concerning physical activity, longer screen exposure, and irregular sleeping patterns [45]. A study concerning Jordanian children and adolescents showed that 70% of the adolescents and 40% of children were spending more than 3 h daily in front of the screen during the lockdown [69]. One additional problem in India was identified by Alvi et al.: school closures resulted in the temporary suspension of mid-day meals and supplementary nutrition programs. Thus, undernutrition and hunger were both possible for students [44].

#### 3.1.2. Youths (15–28 Years Old)

A total of 10,082 youth participants at three educational levels (high school, college, and graduate school) in China reported their lifestyle changes during lockdown. Significant decreases were observed concerning the frequency of intake of rice, meat, poultry, fresh vegetables, fresh fruit, soybean products, and dairy products. It is remarkable that females consumed more rice, fresh vegetables, and fruit and less meat, poultry, soybean, and dairy products compared to males. (Other studies show that it is distinctly more common for women to select less healthy foods when stressed [77]. The economic status is also very important. It was found that in case of an economic crisis, women start to prefer vegetables [78,79]). Furthermore, the frequency of consumption of wheat products, other staple foods, and preserved vegetables was increased. The results showed that males consumed these foods more frequently compared to females [36].

#### 3.1.3. Adults

A total of 22,459 Chinese respondents participated in a study, which indicated that 60.2%, 66.3%, and 66.8% reported impacts on psychology, life, and work respectively. Different psychological impacts led also to different dietary intake (higher psychological impact led to increased dietary intake of salt, fried foods, and sugary foods) [60]. Another study performed in China [70] (2702 participants) showed that 54.3% reported reduced physical activity, 45.5% had increased sleep duration, and 38.2% increased their snack intake. Despite these changes, only 25.0% reported an increase in body weight. Similar results were obtained in Israel. In a study conducted by Kaufman-Shriqui et al. (1895 participants), the authors reported that although approximately 60% indicated that their pre-pandemic diet was healthier than their current diet, only 25.2% indicated that they had gained weight during the COVID-19 pandemic [72]. Moreover, isolation significantly increased the odds of at least mild anxiety. An online survey in Saudi Arabia showed a more sedentary lifestyle and altered eating habits by some residents [73]. Another study including 638 Saudi women reported the following negative results: 42.8% reported depression, 27% anxiety, 71% moderate stress, and 12.5% severe stress. In addition, 40.4% were “moderate” and 12.4% “high” emotional eaters [15]. Data from 506 households in Saudi Arabia showed that COVID-19 had the most detrimental effect on feelings and emotions and the second-most affected domain was physical health and social status of life [56].

Furthermore, an important survey in Jordan (4388 participants, mainly adults, plus 673 students) indicated a significant increase in body weight as well as an increased number of snacks, number of main meals, and smoking rate. In addition, water intake was reported below the recommended in all weight groups [35]. A different survey in Jordan (3129 participants) associated severe food insecurity during lockdown with two key factors: being 18–30 years old and living in a rented house [39]. A total of 1959 adult respondents in Japan participated in a study, which led to the conclusion that thoughts and behavior concerning food waste differ significantly depending on sociodemographic characteristics (gender, household size, and employment status) [74].

The Asian country that counts the greatest number of COVID-19 cases as well as the greatest number of deaths due to COVID-19 is India. A survey in India (including 995 participants) came to the following conclusions. Notwithstanding the fact that during the COVID-19 period the participants improved their eating behavior, one-third of participants gained weight as their physical activity was reduced and screen and sitting time were increased. One fourth of the participants reported an increase in stress as well as anxiety level derived from fear of getting infected by coronavirus, loneliness, boredom, and financial loss at work [19]. Interestingly, a study in Vietnam (8291 outpatients) showed that healthy dietary intake potentially modifies the negative effect of lockdown on depression [75]. A total of 555 Nepalese participated in a survey that led to the conclusion that people developed stress due to the pandemic (76.4%). Of these, 63.6% were adversely affected in their profession or declared economic loss in business. Meanwhile, 42% ameliorated their skills and knowledge (online classes, personal development etc.) [51]. The national measures in Thailand reduced the cumulative min of work-related physical activity, as well as transportation, and recreational physical activity [64]. A lot of conclusions were derived from a survey in the United Arab Emirates. A total of 1012 participants were recruited. Of these, 31% reported weight gain and 72.2% had less than eight cups of water per day. Participants’ dietary habits were not desirable (“unhealthy” dietary patterns). In addition, 36.2% declared they spent five hours daily on screens (not for work). Unfortunately, 60.8% of the participants had sleep disturbances during the pandemic [28].

A total of 1103 adults participated in an online survey, which showed that quality of life had significantly deteriorated among specific categories of people. Specifically, it was reported that the elderly and people with lower income, low educational levels, chronic disease, and concern about COVID-19 infection declared deterioration of quality of life [71].

### 3.2. Europe

Nowadays we know that SARS-CoV-2 arrived in Europe in December 2019, when there was a patient infected in France [80]. To date, more than 215.2 million coronavirus cases have been reported in Europe, with France, Germany, the United Kingdom, Russia, Italy, and Spain being the countries with the highest numbers of cases (in that order) [4]. Accordingly, European countries have been implementing myriad virus-control measures, including mass quarantine (lockdown/confinement). Spanish and Italian governments implemented very strong restrictions at the beginning of the pandemic and therefore many studies about the effects of lockdown on the diet and lifestyle of the general population have been performed in these countries. Table 3 briefly summarizes these studies.

A healthy dietary pattern and adequate physical activity should be seen as a way to prevent, manage, and cure lifestyle-related chronic diseases, thereby promoting health and healthy aging. For example, concerning physical activity, before the pandemic a project was designed to implement exercise as part of routine clinical care in the Netherlands, and it has been continuing as far as possible during the pandemic [123].

#### 3.2.1. Children and Adolescents

Governmental measures included school closures in many countries, therefore affecting children and family routines. A study performed in Denmark included different parameters to assess the effect of lockdown in children from families with different socioeconomic statuses [61]. Remarkably, the authors analyzed reading habits during the initial lockdown (i.e., closed schools, but online teaching) and during the second lockdown (i.e., emergency teaching on a limited schedule) by assessing the use of an application that provides unlimited access to books. They concluded that all children increased their time spent on reading during the initial lockdown. Moreover, this increase was sharper among children of college-educated parents and children of parents above the median income level. Indeed, children of college-educated parents spent more time reading during the entire lockdown period, although there was a general drop during Easter holidays. Unexpectedly, during the second lockdown, reading activity diminished and no clear differences attributed to families’ socioeconomic statuses could be observed. In addition, there were not obvious differences between girls and boys during the whole lockdown period [61].

Regarding nutrition and physical activity habits, a survey answered by parents of Spanish children and adolescents (aged 3–16) showed that daily fruit and vegetable consumption and physical activity were reduced during confinement, whereas screen exposure was increased. Counter-intuitively, sleep time tended to slightly increase [81]. A longitudinal study in Spanish children and adolescents (aged 8–16) detected similar negative results regarding screen exposure and physical activity, which was even more reduced in children of non-Spanish or non-university-educated mothers [82]. In contrast to the previously mentioned study [81], the authors described an improved adherence to a Mediterranean diet [82].

This decrease in physical activity has been observed in other European countries. Remarkably, only 20% of Irish adolescents (aged 12–18) were more physically active during lockdown than before. In contrast, around half of the adolescents reported a decrease in physical activity. This group included a higher percentage of overweight adolescents and adolescents with obesity, as well as subjects with less strong prior physical activity habits. When asked about barriers to physical activity, adolescents mentioned coronavirus, club training cancellations, and time, while they said that more time, coronavirus, and lack of school were facilitators for physical activity [83].

Nowadays, obesity is an important health problem among children and adolescents, and it is increasing due to COVID-19-related mobility restrictions [124]. Indeed, children and adolescents with obesity from Italy increased their intake of potato chips, red meat, and sugary drinks, as well as their fruit intake, whereas their vegetable consumption remained the same. Furthermore, they spent less time playing sports and more time sleeping and watching screens. These changes in their lifestyles will undoubtedly worsen their obesity problems [84]. In contrast, another study in Italian adolescents (aged 15–18) showed that, although lockdown had negative effects on inactive and moderately active students by decreasing even further the time spent on physical activity, highly active students increased the time spent on physical activity during and after lockdown, compared to the previous situation [85].

Notably, an Italian study showed negative and positive effects of lockdown [24]. Italian adolescents (aged 15–21) found it hard to be at home and it affected their physiological well-being, including anxiety. Nevertheless, they developed new interests and planned their daily routines in a different way, such as engaging in physical activity, cooking, playing videogames, reading, and playing board games. Most of the adolescents kept in touch with their friends and, if engaged in a romantic relationship, they reported missing the support of their partners. Although there was a significant increase in family quarrels, many adolescents were more prone to express their feelings to their parents. Moreover, their sleep-waking cycle did not generally change (although many of them were going to bed later). Therefore, although many adolescents experienced worsened subjective well-being perception, with possible implications for their mental health, some positive effects were noticed [24]. Other health problems associated with lockdown were chilblains in predisposed Italian adolescents (aged 11–15), due to cold exposure [86].

During an Austrian survey, apprentices reported significantly more smoking than high school students and this difference was more pronounced in women. Alcohol consumption was higher in apprentices than school students, but only in women [91].

In conclusion, European children and adolescents faced a new daily routine and adapted by acquiring both good and bad habits.

#### 3.2.2. University Students

In many European countries, universities were closed and/or online teaching was implemented. These measures led to problematic eating behaviors in university student populations, such as binge eating. In a sample of French university students, binge eating was more frequent among overweight students or students with obesity, compared to those of a healthy weight, and in females compared to males, and was associated with high levels of stress (emotional eating). On the other hand, stress and anxiety were also associated with dietary restriction. Students at risk of eating disorders were prone to experience aggravation of these conditions during lockdown [25].

Food procurement and nutritional habits were assessed in students from Bavarian universities, in Germany, and it was concluded that the percentage of home-cooking did not change significantly, while cafeteria and restaurant food consumption decreased, since these establishments were closed. Overweight participants more frequently increased their amount of food consumption. The subgroup that reported to be eating more during lockdown than before also mentioned eating more confectionary and bread, while no changes in other categories of food were observed in the whole sample [54].

As expected, due to mobility restrictions, most Italian medicine students reduced their total physical activity. Overall, they were more sedentary, slept more, and walked less during lockdown. Although they increased their moderate and vigorous activity, this habit would probably not have been enough to counteract the negative effects of a general decrease in physical activity [47]. Another study analyzed Italian university students and reached similar conclusions: participants of this study also reduced their total physical activity, especially by reducing walking time, and increased sedentary behavior during lockdown, particularly by increasing time of use of electronic devices. Being previously active, female, and younger than 22 years old, among other factors, correlated positively with the probability of reaching the recommended levels of physical activity during lockdown [88]. Another study in students from Bavarian universities focused on their physical activity; almost half of the students indicated a decrease in physical activity caused by lockdown implementation, whereas one third was training more during lockdown than before. Moreover, and unexpectedly, a general decrease in walking (steps) was observed [87]. In a more thorough study on Spanish university students, the authors also concluded that sedentary behavior (sitting hours) increased during lockdown, but, contrary to the aforementioned studies, they also reported an increase in physical activity [90].

Finally, a study on Italian university students wanted to unveil the relationship between diet, exercise, and physiological state. They found a direct association between physical activity and fruit, vegetables, and fish intake, as well as an association between cereal, legume, and low-fat meat consumption and both depression (positive association) and quality of life (negative association). These data support the idea of a vicious circle between poor mood states and bad nutritional habits, the first leading to the second one and vice versa. Physical activity can be the key to stop this vicious circle, due to its promotion of healthy nutritional habits, and, therefore, better mood states during lockdown [59]. Furthermore, according to the EPICO Study, performed at the very beginning of the pandemic, although most Italian undergraduate students reduced their physical activity, those enrolled in life sciences courses showed a higher awareness regarding the virus and the control measures [89].

In summary, lockdown had both detrimental and beneficial effects in the daily life of students, and some subpopulations adapted in a healthier way to the new situation.

#### 3.2.3. Families

Family and the roles of each member have been disrupted by the new challenges of the pandemic, such as school and restaurant closures, leading to an increase in domestic responsibilities and childcare. In this line, new mothers in the United Kingdom indicated that their partner, health professional, and online groups were the most important sources of support for infant feeding. Many of them said that they were missing emotional support after giving birth, as well as support for their own health. Overall, the pandemic led to increased mental health problems in new mothers [92].

An interesting study in Iceland, a leading country in gender equality, revealed that Iceland’s semi-lockdown (reduction in school hours) due to COVID-19 intensified the tensions of the neoliberal idea of parenthood, i.e., the perfect and optimistic parent who copes with children and paid work, and uncovered the feminist insight that mothers were forced to deal with housework, childcare, and being ultimately responsible for their children’s academic success in a much more demanding way than fathers. Some of the participants expressed their hopes that the COVID-19 semi-lockdown would improve gender equality [57].

#### 3.2.4. Adults

A compelling amount of data indicates that adults (the general healthy population) have been deeply affected by governmental restrictions aimed at stopping the spread of COVID-19 and health systems’ collapse. A study from Spain identified worsening dietary patterns, and mental health, unemployment, or negative changes in the work situation, expected economic problems, and anxiety about a relative’s possible infection as the major stressors linked to increased risk of depressive episodes [95]. Another Spanish sample showed increased weight, decreased physical activity, and worsening of sleep problems and self-perceived well-being, the latter being more intense in more populated households [11]. Similar results were obtained in Croatia: a decrease in physical activity and The number was corrected The number was corrected increase in body weight (sharper in women and high body mass index individuals), as well as frequent sensations of fear, discouragement, and sadness [22]. In one study, Italian adults also experienced weight gain, decreased physical activity and sleep problems [23], but another study noted positive and negative changes in exercise, sleep, food, and tobacco consumption [102]. The Polish population, which was very well informed about symptoms and prevention of COVID-19 infection from the very beginning of the pandemic, became more afraid of the pandemic and the subsequent expected economic crisis, as well as how all these facts could affect their families [20]. Indeed, Polish adults reported deaths of loved ones, severe COVID-19 disease in loved ones, healthcare failures, and individual and social consequences of the pandemic as their most important concerns, and experienced a decline in happiness and life satisfaction [21]. A similar decline was observed in members of the Irish Men’s Sheds Association, as well as a decline in physical activity and stronger feeling of loneliness [16]. The Spanish population also reduced their physical activity (including vigorous physical activities and walking time) and increased sedentary behaviors [46]. Similar results were perceived in an Italian sample, where responders decreased their total physical activity during lockdown, and this decrease impacted negatively on psychological health and well-being [17].

Conversely, an increase in physical activity was observed in Belgian adults during lockdown. Nevertheless, enhanced sedentary behavior was also observed. Previously less-active adults younger than 55 years old had been exercising more. However, previously active individuals above 55 years old, with a lower education level, and who were not using online physical education tools and practicing sports in groups or in a sport club were undertaking less physical activity. Their reasons included increased sitting hours, lack of time, and absence of the social and competitive elements of sports [12].

In a study in Poland there was a trend towards decreased physical activity and increased screen time among almost half of the responders, and an increase in food consumption in a third of the sample. Many participants reported a prohealthy change in their dietary patterns, but others said that their diet become unhealthier during lockdown [107]. Indeed, Polish adults’ dietary patterns changed during lockdown: they were snacking more and eating more meals per day. They were consuming more eggs, potatoes, sweets, canned meat, and alcohol but less fast food, instant soups, and energy drinks. Consequently, two thirds of the participants in this study experienced body weight changes during lockdown and half of them were overweight [34]. Dutch adults [112], United Kingdom adults [93], as well as Italian adults [103] who were overweight or obese had unhealthy eating habits (chips, snacks, non-alcoholic beverages, and so on) compared to those of a healthy weight. Another study in Polish adults confirmed this trend towards more snacking and eating, especially among overweight subjects and subjects with obesity. Weight gain (mainly in individuals who were overweight or obese, and older individuals) and weight loss (mostly in underweight individuals) were both reported. As expected, the authors found a negative association between body mass index and vegetable, fruit, and legume consumption, and a positive association between body mass index and consumption of fast food, meat, and dairy products. Many alcohol addicts and smokers increased their consumption of alcohol and tobacco, respectively, during lockdown [108]. The French population also changed addiction-related habits, in a negative or positive way, although more negative changes were observed regarding alcohol, tobacco, and cannabis use, caloric/salty food intake, and screen exposure [50]. In an Italian study, almost half of the responders reported weight gain and around 40% mentioned a slight increase in physical activity, and only 3.3% of smokers had quit smoking during lockdown [104].

Lithuanian adults also changed their lifestyle during lockdown: almost half of the participants in one study were eating, snacking, and cooking at home more often. Similar to the Polish sample [34], they were consuming less fast food, carbonated or sugary drinks, and commercial pastries, whereas they were eating more homemade pastries and fried food. The majority of them reduced their physical activity during lockdown, with more than one third of the participants gaining weight [114]. More than one third of a sample of Polish women gained weight, mainly due to reduced exercise and increased food intake and screen time, whereas almost one fifth of them reduced weight [109].

Another finding from Poland was that those living in villages and towns below 5000 inhabitants exhibited a trend to inactivity before the pandemic but enhanced their physical activity during lockdown. On the contrary, residents of large cities showed a weak tendency to be less active during lockdown. Furthermore, beliefs and behaviors related to dietary supplements, medication, and health were not affected by the pandemic in the general population of Polish adults [110].

Remarkably, dietary habits affect and are affected by physiological health. Almost half of the participants in a study in Italy were eating more during lockdown, with an increase in consumption of chocolate, ice-cream, desserts, and salty snacks (“comfort food”). Most of them attributed these variations in their diet to anxiety, stress, or boredom, and only a few subjects referred to a greater difficulty in finding certain products. Consumption of fruit and vegetables remained similar for most of them, but those who decreased their consumption said that it was due to a reduced interest in eating them, although some people found it difficult to buy them [62]. A high percentage of Italian adults participating in another study were experiencing depressed mood, hypochondria, insomnia, and anxiety. In many cases, this anxiety was due to their eating habits and led to the consumption of “comfort food”, especially in female and older individuals [18]. Regarding anxiety, a Portuguese study found that females and people aged 18–34 were the subgroups of adults that tended to be more anxious during lockdown [115]. An increase in bad mood was also observed in Scottish adults, and it was linked to poor diet and sleep quality, and low physical activity, among other factors [116].

In addition, around half of the participants in a French study were feeling more depressed, stressed, or irritable since lockdown was established. A higher percentage had increased their alcohol and tobacco consumption, although some people reduced their intake. Nevertheless, almost a third of the individuals had changed their nutritional habits towards a more balanced diet, whereas only 17% said that their diet was less balanced due to lockdown [113]. Data from a Spanish population also indicate a positive variation in their dietary pattern: more fruits and vegetables and less processed food, even though they were eating more and snacking more often than before lockdown [96]. Another Spanish study identified several changes in diet and substance use, as well as strategies to deal with stress, sleep pattern, and social support (around one third of the participants), physical activity (>70% of the participants), and patterns of indoor/outdoor time (>90%) [97].

With regards to specific healthy diets, the majority of the Italian adults and their children with celiac disease participating in a study maintained their gluten-free diet during lockdown, and one third of the responders even improved adherence to this specific diet [105]. Data about Italian adults pointed to maintained adherence to a Mediterranean diet, particularly in the group aged 18–30 [104]. Better adherence to a Mediterranean diet was identified in Spanish adults [98,99], similar to that observed for children and adolescents [82], but an increase in homemade desserts and pastries and a decrease in the number of subjects exercising and the hours spent on exercise were also observed [99].

A study that analyzed adult samples (15–82 years) from several European countries (Bosnia and Herzegovina, Croatia, Greece, Kosovo, Italy, Serbia, Slovakia, Slovenia, and Spain) concluded that increased physical inactivity (less walking and sports) and greater sleep and screen exposure led to weight gain. Despite this, lockdown had positive effects on nutrition: meals were more regular and healthier and alcohol intake and smoking decreased [117]. Adults (15 and older) from Norway and Sweden were both eating more and being more sedentary, albeit at different rates [118].

Food insecurity or fear of food shortages, even if they do not happen, also affected the European population. Being afraid of food shortages during lockdown was associated with eating the same or a reduced amount of fruit and vegetables, and it was also associated with drinking the same or more soft drinks, according to data from Belgium [111].

Last but not least, the environmental impact of changes in nutritional patterns should be considered. In Spain, lockdown led to diet alteration towards higher energy intake and lower nutritional quality, therefore increasing the global warming potential, land use, and blue water footprint [100].

However, there are some optimistic data regarding adaptative capacity. All health risk behaviors (related to physical activity, alcohol consumption, fresh fruit and vegetable consumption, smoking, screen exposure, and sleep hours) were increased at the beginning of the lockdown but then all of them, except screen exposure, decreased in a time-dependent fashion during the first three weeks of lockdown, according to data from a Spanish sample. Straightforward interpretation of these results suggests an adaptation of the Spanish adult population to the new context [27]. Additionally, pets provided emotional support to their owners, but the animals also showed signs of stress [101].

Among adults, a particularly vulnerable subgroup is the population with previous mental health problems. Indeed, Spanish patients with a psychiatric disorder indicated more difficulties in dealing with the stress associated with the COVID-19 pandemic and lockdown [26]. Moreover, United Kingdom adults with current or past eating disorders, especially women, had more problems in regulating eating and were more concerned about body image [94]. Portuguese patients with eating disorders also experienced a worsening of these conditions during lockdown [119]. Furthermore, Italian patients with neuromuscular disease, as well as healthy controls, practiced less physical activity during lockdown [106].

Age is also an important factor in developing strategies to deal with lockdown. A Spanish study demonstrated that each generation had a different response to the new situation, with the youngest group (aged 18–33 years) having more problems in maintaining a daily routine, more hyperactivity and sleep problems, and increased depression, anxiety, and stress, and older adults (aged 46–60) being more able to adapt to the lockdown [14].

Thus, it is difficult to draw a general conclusion about European adult populations, since different subgroups were able to deal in a better or worse way with the pandemic.

#### 3.2.5. Older Adults

Unhealthy lifestyles during the COVID-19 pandemic increase the risk of non-communicable diseases, therefore impeding healthy aging [125]. Nevertheless, we already mentioned that older Spanish adults (aged 46–60) had reduced sleep problems, depression, anxiety, and stress symptoms [14]. Likewise, older adults in the Netherlands (aged > 65) were more capable of maintaining their eating behaviors during lockdown, compared to younger adults, maybe due to the fact that they were not in the labor market, so they were not affected by changes in their working schedule or jobs loss [112]. Nevertheless, another study among older adults (aged 62–98) showed that around one third of the participants changed their eating habits in a way that predisposed then to overnutrition, for example by snacking more than before lockdown implementation, and that around half of them decreased their physical activity [120]. Furthermore, older adults in France (>60) reduced their participation in group physical activities because of the pandemic but expressed their desire to exercise at home [121]. Regular exercise positively correlated with self-efficacy and optimism and negatively correlated with depressive symptoms in Spanish older adults (aged 60–92) [122].

Summarizing, the effects of confinement and restrictions associated with the COVID-19 pandemic on the European population are large and varied. Despite having some positive effects in some subgroups (increased physical activity and enhanced adherence to a Mediterranean diet, among others), the overall outcome is mostly pessimistic. Long-term effects on physical and mental health of European inhabitants are unknown but programs aimed at promoting healthy habits, such as exercise and healthy diets, should be implemented, as well as psychological support and follow-up strategies to evaluate the efficacy of these programs.

### 3.3. North America

To date, the official number of COVID-19 cases in the whole region of the Americas (North, Central and South America, as well as Caribbean and Atlantic Ocean Islands) is 182,429,670. In North America the number is 92,825,957, with the United States of America (831,888,551 coronavirus cases) having the higher number of cases [126]. Despite this, few studies (summarized in Table 4) are available about the effects of confinement on these populations’ nutritional habits, physical activity, and other lifestyle parameters.

#### 3.3.1. Children, Adolescents, University Students and Families

School closures had consequences for North American children and adolescents’ daily life. Since many families in the United States were relying on meals (breakfast and lunch) served in schools, food insecurity and the risk of undernutrition increased for these children, mostly in low-income families. The United States government launched many programs aimed at solving this issue [38]. Importantly, food insecurity in the United States households with children was higher in low education neighborhoods, low-education families, and disadvantaged ethnicities (particularly non-Hispanic blacks) [127].

Around one fifth of university students were also experiencing food insecurity, according to data from the United States. On the contrary, and expectedly, students who moved with their families during the pandemic or received financial support improved their food security [128].

With respect to physical activity, at the beginning of the COVID-19 pandemic, most children performed exercise by free playing and walking, and they conducted this at home indoors or on neighborhood streets more often than in pre-COVID-19 times. One third of them followed activity lessons, but in general they spent many hours per day engaged in sedentary leisure activities. The increase in sedentary behavior and decrease in physical activity were sharper in older children (aged 9–13), when compared to younger children (aged 5–8) [129].

Similar results were observed for Canadian children and adolescents: reduced time spent outside, less physical activity and more sedentary behavior (such as leisure) during the pandemic. An increase in sleep was also described. Children and adolescents whose parents were performing physical activity and/or encouraging them to do so, as well as those owning a dog, were more prone to be active. The optimistic result was that many parents were looking for new hobbies and accessing resources to better cope with the new situation [130]. Another study in middle- to high-income Canadian families confirmed previous results (less physical activity and more sedentary behavior, in this case related to screen time) and also concluded that families were cooking more at home and, at the same time, snacking more. Their main sources of stress were work–childcare balance and economic instability [58].

#### 3.3.2. Adults

The majority of a United States population sample spent more time at home due to COVID-19 and self-quarantine guidelines. Around 20% of the studied population gained weight and this increase in body weight was associated with decreased physical activity, unstructured and emotional eating, snacking after dinner, and lack of sleep [131]. Obesity was more extended in areas where adults followed more often unhealthy dietary patterns in the pre-COVID-19 period and these patterns incremented during the pandemic [132]. In another study in the United States, responders indicated no changes in their dietary patterns, but increased consumption of sweets and salty snacks. In these sample, food attitudes (including eating much more than planned, over-eating, lethargy after eating, and stress behaviors) were associated with food security [133].

The same factors (decreased physical activity and increased screen exposure) were observed in another study and were related to stress, poor mental health, depressive mood, and loneliness [134]. Canadian adults, especially younger, Canada-born adults and individuals who were financially affected by the COVID-19 pandemic, also showed negative health outcomes: increases in screen time and alcohol and junk food consumption [136].

Previously active Canadian individuals were less prone to decrease their physical activity during the pandemic than previously inactive subjects. The previously inactive population’s well-being was more affected by their physical activity levels during the pandemic, exercise being a source of social, emotional, and psychological health. Consequently, outdoor physical activity was helpful to lower anxiety in previously inactive individuals [137].

Canadian women were more affected by the pandemic, since they had more difficulties to maintain their pre-COVID-19 levels of physical activity and they experienced more anxiety than men [13]. Female twins from the United States also had more anxiety than males, while older twins tended to have less anxiety. Additionally, almost half of the twins participating in the study had decreased their physical activity during the pandemic [135].

In conclusion, in a comparable way to European countries, the North American population has been mostly negatively affected by confinement in terms of nutrition, physical activity, and psychological health.

### 3.4. South America

Regarding South America, Brazil is the country which counts the greatest number of COVID-19 cases (30,977,661) and deaths [126]. Therefore, many surveys in Brazil (related to COVID-19) have been published. A brief summary of studies related to the consequences of the lockdown in South American populations is presented in Table 5.

#### 3.4.1. Families with Children Aged Less Than 13 Years

A survey included the responses related to 816 children aged from zero to 12 years. The parents declared a reduction in the levels of physical activity among their children. Of all respondents, 38% reported also that screen time is higher than in regular school hours, and 36.9% indicated this was much higher. Interestingly, 52.1% claimed to have more family activities than before isolation [138].

#### 3.4.2. Adults

Brazilian adults changed their lifestyle during lockdown. An important survey with 45,161 individuals sheds light on various lifestyle changes. First of all, there was a decrease in physical activity. Moreover, an increase in screen time, intake of ultra-processed foods, smoking, and alcoholic beverage consumption were also observed. Notably, 34% of smokers reported an increase in their cigarette consumption. The highest prevalence of alcohol consumption (24.6%) was observed among people aged 30–39 years old. In addition, the survey indicated that the greatest proportional increase in consumption of all unhealthy foods was typical among young adults (18–29 years old) [49]. Similar results were published by Werneck et al. [139]. The main conclusion was that Brazilian adults with sedentary behavior were also more likely to present an unhealthy diet lifestyle during quarantine.

Another study assessed the association between previous diagnoses of depression and changes in health behaviors during the COVID-19 lockdown. Participants with depression were more likely to present an elevated frequency of ultra-processed food consumption [140].

In Brazil, the Crisis Committee (CC-AGRO-COVID-19) was formed in order to analyze production, marketing, infrastructure, social perceptions, and agricultural products in relation to the pandemic. Particularly, Brazil tried to identify potential risks and prevent a severe crisis due to the COVID-19 pandemic [40]. The government of Brazil focused on promotion of supply and structuring of sustainable and decentralized systems with an agroecological base concerning food production, extraction, processing, and distribution [40]. Unfortunately, The Food and Agriculture Organization (FAO) estimated that the COVID-19 pandemic would be fatal as the expected number of people in food insecurity by 2020 would increase from 135 million to 265 million [52]. A study conducted in Brazil by UNICEF and the Brazilian Institute of Public Opinion and Statistics (IBOPE) indicated that during COVID-19, 33 million adult Brazilians experienced an instance of having no money to buy food. Furthermore, in the same study, it was reported that about nine million Brazilians were unable to have a meal. The main reasons were (i) there was no food and (ii) there was no money available to buy the meal [29].

A survey conducted in Chile aimed to discover positive and negative changes in food habits, physical activity patterns, and weight status. Different factors had a positive association with body weight increase: sedentary time ≥ 6 h/day, low water consumption, and consumption of fried foods ≥ 3 times per week. Moreover, a middle socioeconomic background was associated with body weight increase. Daily alcohol consumption was associated with physical activity decrease [37].

A total of 1022 adults in Ecuador participated in a survey aimed at finding out information related to eating habits and sleep quality during the COVID-19 pandemic. The following results were obtained: the population decreased their physical activity during the COVID-19 lockdown, both in high and moderate intensity, while low intensity became predominant during the same period. Interestingly, the population improved to a certain extent their eating habits. At the same time, it was observed that unhealthy habits were reduced [141].

Another survey in Ecuador (including 9522 Ecuadorian adults) resulted in important findings. Firstly, sleep quality differed according to sex (it was worse in women). Secondly, women had greater changes in the habitual consumption of food when compared to men. Furthermore, people 18–40 years of age decreased their food consumption compared to people > 40 years [142].

### 3.5. Oceania

The first confirmed case of COVID-19 in Oceania was announced on 25 January 2020 by Victoria Health Authorities [143]. To date, more than 8.3 million coronavirus cases have been reported in Oceania [4]. The consequences due to COVID-19 are discussed below. A brief summary of studies related to the consequences of the lockdown in Oceanian populations is presented in Table 6.

#### 3.5.1. University Students

An Australian study of university students showed that, in females, the energy intake was approximately 20% greater during the COVID-19 period, and snacking frequency and energy density of consumed snacks increased (compared with the two previous years). Physical activity changed for both sexes during the pandemic. Approximately 30% fewer students reported following a lifestyle with sufficient levels of activity [144].

#### 3.5.2. Australian Young People (Aged 16–25)

An online survey with respondents in Australia showed elevated levels of depression and anxiety. Social media use was high as a result of the pandemic [145].

#### 3.5.3. Adults

Respondents reported decreased enjoyment of grocery shopping. At the same time, they increased home cooking as well as baking. Unfortunately, there was an overall increase in unhealthy dietary patterns, e.g., sweet snacks, salty snacks, alcohol, and sugary drinks, during the period of the COVID-19 lockdown [48].

### 3.6. Africa

On 14 February 2020, the first case of COVID-19 was confirmed in Egypt by the Minister of Health and Population of Egypt [146]. To date, more than 11.6 million coronavirus cases have been reported in Africa, with South Africa, Morocco, and Tunisia being the countries with the highest number of cases (in that order) [147]. A brief summary of studies related to the consequences of the lockdown in African populations is presented in Table 7.

#### 3.6.1. Adults

An online survey with respondents in two East African countries (Kenya and Uganda) assessed the implications of COVID-19. The conclusions derived from the survey were that (i) food security worsened and (ii) the dietary quality of respondents worsened. Losses and reductions in income, as well reduced access to markets, were observed [41].

#### 3.6.2. Low-Income Rural Households

During a survey (via weekly interviews in Kenya) a decreasing trend in total cash inflows and outflows was observed for low-income rural households. The timeframe of this research was the period March–April 2020. Income from work as well as receipts of gifts and remittances decreased [148].

### 3.7. Surveys Conducted in Many Countries

An online survey including 5896 respondents conducted in 17 countries of the Middle East and North Africa (Egypt, Jordan, United Arab Emirates, Kuwait, Bahrain, Saudi Arabia, Oman, Qatar, Yemen, Syria, Palestine, Algeria, Morocco, Libya, Tunisia, Iraq, and Sudan) resulted in the following conclusions: the pandemic was associated with an increase in food consumption and sedentary lifestyles [149]. These findings are similar to the aforementioned Asian studies.

It is worth mentioning that similar trends during the pandemic (parameters in which the same effects were observed in different countries) were recorded in this review. The most frequent effects are as follows: (i) reduced physical activity (reported in 4 references derived from Asia, 19 references from Europe, 6 references from North America and 3 from South America), (ii) weight gain (reported in 5 references derived from Asia, 7 references from Europe, 1 from North America, and 1 from South America); and (iii) increase in screen exposure (reported in 4 references derived from Asia, 7 references from Europe, 2 from North America, and 2 from South America). The distribution worldwide of these three different effects are presented below (Table 8).

## 4. Conclusions

The effects due to lockdowns are many and encompass a variety of aspects of daily life worldwide. The COVID-19 lockdowns resulted more often in negative consequences (described in Table 1) and, rarely, in positive consequences (occasionally healthier cooking at home, increased time with family, or development of new interests and different routines (e.g., walking)). Unfortunately, the negative effects have affected lifestyles worldwide. Another significant problem is that the consecutive waves of the pandemic have resulted in economic and psychological exhaustion for many people. As the fourth wave of the pandemic spreads around the world, more studies are needed to better understand all of these effects. New studies should be aimed at larger groups of individuals and utilize questionnaires that are as comprehensive as possible. In addition, the long-term prevalence of the virus necessitates the most accurate government guidelines possible.

## Figures and Tables

**Table 1:** Main effects due to lockdown.

Parameter in Which Effects Are Observed	Effect	Representative Study (Where It Is Described)	Effect Reported in (i) Country (Sample Size) or (ii) Elsewhere e.g., in a Review
**Physical activity**	Decreased physical activity	[11,12]	Spain (161), Belgium (13,515)
**Psychology**	Increased anxiety in women	[13]	Canada (1086)
	Fewer coping strategies in younger adults (compared to older adults)	[14]	Spain (3524)
	More depressed, stressed, or irritable behavior	[15]	Saudi Arabia (638)
	Loneliness	[16]	Ireland (383)
	Poorer psychological health and well-being	[17]	Italy (2974)
	Hypochondria	[18]	Italy (602)
	Increased fears about the disease	[19]	India (995)
	Worries about families	[20]	Poland (2618)
	Decline in happiness and life satisfaction	[21]	Poland (412)
	Discouragement and sadness	[22]	Croatia (3027)
**Consequences on health**	Weight gain	[23]	Italy (121)
	Bad psychological health	[24]	Italy (306)
	Worsening of eating disorders	[25]	France (5738)
	Fewer coping strategies in patients with a psychiatric disorder	[26]	Spain (619)
	Increased health risk behavior	[27]	Spain (2741)
	Sleep problems	[28]	United Arab Emirates (1012)
	Reduced availability and accessibility of health services	[29]	Brazil (1516)
	Micronutrient deficiencies	[30]	Review
**Eating habits and alcohol**	Increased junk food consumption (sweets, salty snacks, etc.)	[31] [32] [33]	Review; Review; Belgium (28,029)
	Increased alcohol consumption	[34]	Poland (312)
	Increased number of main meals and number of snacks between meals	[35]	Jordan (4388)
	Decrease in daily fruit and vegetable consumption	[36]	China (10,082)
	Water intake (reported below the recommended)	[37]	Chile (700)
**Effects on society on nutrition issues**	Risk of undernutrition	[38]	United States (-)
	Food insecurity	[39]	Jordan (3129)
	Decreased food production and limited food transport	[40]	Review
	Market closures causing food shortages	[41]	Kenya (313) and Uganda (129)
	Food safety measures (e.g., washing hands before preparing or eating food, illegal trade in wild animals has been banned, etc.)	[42] [43]	World Health Organization article; Discussion in scientific journal
	Food deliveries have increased	[15]	Saudi Arabia (638)
	Temporary suspension of mid-day meals and supplementary nutrition programs	[44]	Opinion piece in scientific journal
**Lifestyle**	Increased screen time (use of electronic devices)	[45]	China (10,416)
	Increased sedentary behavior	[46]	Spain (3800)
	Increased sleep	[47]	Italy (1470)
	More cooking at home	[48]	New Zealand (3028)
	Smoking	[49]	Brazil (45,161)
	Cannabis use	[50]	France (11,391)
**Economy**	Economic crisis	[51]	Nepal (555)
	Unemployment	[41]	Kenya (313) and Uganda (129)
	Price volatility	[52]	UNICEF article
	Impact on tourism sector	[52]	UNICEF article
	Logistical constraints (international shipping and domestic transport interruptions)	[53]	India (original research)
	Closures of restaurants have affected demand for specific foods	[54]	Gernany (1964)
	Decrease in demand for foreign labour	[55]	International Monetary Fund article
	Decline in employment	[56]	Saudi Arabia (506)
**Consequences on society (apart from nutrition issues)**	Amplification of tensions of parenthood	[57]	Iceland (97)
	Gender relations	[57]	Iceland (97)
	Housework and childcare	[58]	Canada (361)
	Quality of life	[59]	Italy (176)
	Development of new interests and different routines	[24]	Italy (306)
	Implications on productivity	[60]	China (22,459)
	Implications on education	[61]	
	Panic-buying of food and other essential items	[62] [63]	Italy (1932); China (1188)
	Social distancing measures	[52]	UNICEF article
	Restrictions on international transport	[5,6,7]	World Health Organization article; UNICEF article; Qualitative Study on the United States, China, South Korea, the United Kinddom, Brazil, and Haiti
	Protection equipment at work	[60]	China (22,459)
	Remote working	[64]	Thailand (4460)
	Disrupted balance between work and family because of the increase in workload brought by the remote working model	[65]	Review
**Agriculture**	The absence of agricultural labor has affected planting, harvest, and post-harvest operations	[66]	Articlo for Asian Productivity Organization Members *
	Shortage of inputs (fertiliser, seed, feed, and/or pesticides)	[66]	Article for Asian Productivity Organization Members *

* Asian Productivity Organization Members: Singapore, Japan, Malaysia, Republic of Korea, Turkey, Thailand, Vietnam, Indonesia, Philippines, Sri Lanka, India, Pakistan, Nepal, Bangladesh, Cambodia, The Lao People’s Democratic Republic.

**Table 2:** Brief summary of studies related to the consequences of the lockdown in Asian populations.

Population Studied	Sample Size (N)	Methodology of the Study	Impact/Changes Observed during COVID-19 Pandemic	Reference
Chinese students	10,416	Online survey (structured questionnaire through WeChat)	(i) 58.7% decreased participation in physical activity (ii) 76.9% reported longer screen exposure (iii) 38.5% suffered from irregular sleeping pattern	[45]
Jordanian children and adolescents	477	Online survey (structured validated questionnaire built using Google Form)	70% of the adolescents and 40% of children reported spending more than 3 h daily in front of the screen during the lockdown	[69]
Youths (15–28 years old)	10,082	Online survey (questionnaire through chat groups and/or moments on WeChat and Tencent QQ)	Females consumed more rice, fresh vegetables, and fruit and less meat, poultry, soybean, and dairy products compared to males. Furthermore, the frequency of consumption of wheat products, other staple foods, and preserved vegetables was increased. Males consumed these foods more frequently compared to females	[36]
Chinese adults	22,459	Online survey (structured questionnaire with close-ended questions in a WeChat public account)	60.2%, 66.3%, and 66.8% self-reported impact on psychology, life, and work, respectively. Different psychological impact led also to differences in dietary intake	[60]
Chinese adults	2702	Online survey (conducted via the questionnaire platform “Wenjuan xing” and distributed via WeChat)	38.2% increased their snack intake, 54.3% reported reduced physical activity, and 45.5% had increased sleep duration. Only 25.0% reported an increase in body weight	[70]
Chinese people	1139 (1103 adults and 36 < 18 years old)	Online survey (questionnaire distributed via WeChat)	Quality of life had significantly deteriorated among the elderly and people with lower income, low educational levels, chronic disease, and concern about COVID-19 infection (mean EQ-5D index score of 0.949 and a mean VAS score of 85.52)	[71]
Israel adults	1895	Online survey (The survey was uploaded to both public and personal social media sites)	Almost 60% indicated that their pre-pandemic diet was healthier than their current diet, and 25.2% indicated they had gained weight during the pandemic. A worsening of diet quality during the pandemic, weight gain, and isolation significantly increased odds of at least mild anxiety	[72]
Saudi adults	1965	Online survey (survey designed by multidisciplinary scientists and academics, shared through the Google platform)	More sedentary lifestyle (21% vs. 22.9%) and altered eating habits (snacking: 27.4% vs. 29.4%, and no fresh fruits and vegetables: 2.4% vs. 3.7%) by some residents of Saudi Arabia	[73]
Saudi (adult women)	638	Online survey ((i) questionnaire cascaded to different social media, (ii) statements in Likert scale format)	42.8% reported depression, 27% anxiety, 71% moderate stress, and 12.5% severe stress. In addition, 40.4% were “moderate” and 12.4% “high” emotional eaters	[15]
Saudi households	506	Online survey (WhatsApp, Facebook, and LinkedIn, were used to circulate the survey and collect data).	COVID-19 had the most detrimental effect on the domain of affection (such as feelings and emotions), and the second-most affected domain was physical health and social status of life.	[56]
Jordanian adults (and some students)	4388 (673 students)	Online survey (standardized and validated structured questionnaire distributed through social networking sites (SNS), mainly Facebook)	Significant increase in body Weight (12.9% underweight, 28.5% normal body weight, 36.4% overweight’ and 41.1% obese). Increased number of snacks, number of main meals, and smoking rate. Water intake was reported below the recommended in all weight groups	[35]
Jordanian adults	3129	Online survey (a validated questionnaire endorsed by “The Crown Prince Foundation (CPF)”, Ministry of Health (MOH), Ministry of Higher Education (MOHE) and mainly, Hashemite University, and the University of Petra)	Association of severe food insecurity during lockdown with low income, age 18–30 and living in a rented house	[39]
Japanese adults	1959	Online survey (survey through Yahoo! Crowdsourcing service-structured questionnaire consisting of 29 question items)	Thoughts and behavior concerning food waste differ significantly dependending on sociodemographic characteristics	[74]
Indian adults	995	Online survey (validated questionnaire using Google Forms web survey platform and telephonic interview)	One third of participants gained weight as their physical activity was reduced and screen and sitting time were increased. One fourth of the participants reported an increase in stress as well as anxiety level derived from fear of getting infected by coronavirus, loneliness, boredom, and financial loss at work	[19]
Vietnamese adult citizens—outpatients	8291	Recruited at outpatient departments (OPDs) from 15 hospitals and 3 health centers across Vietnam	Healthy dietary intake potentially modifies the negative effect of lockdown on depression	[75]
Nepalese adults	555	Online survey (structured questionnaire in Google Forms)	People developed stress due to pandemic (76.4%). 63.6% were adversely affected in their profession or declared economic loss in business. Meanwhile, 42% ameliorated their skills and knowledge (online classes, personal development etc.)	[51]
Thai adults	4460 Thai adults from Thailand’s Surveillance on Physical Activity (SPA) 2019 and 4482 respondents from SPA 2020	Face-to-face interviews vs. online questionnaires	The national measures in Thailand reduced the cumulative mean of work-related physical activity (74.6% vs. 54.7), as well as transportation and recreational physical activity	[64]
Middle-Aged Korean Workers	12,621	1:1 interview using a laptop equipped with a survey program	There were three types of latent classes. 22.1% of the total participants reported “decrease in all health behavior type”, 51.9% admitted “fast food and delivered food type” increase, and 26.1% reported “increase in smoking maintenance type”	[76]
United Arab Emirates	1012	Online survey (distributed using email invitations and social media platforms, e.g., LinkedIn, Facebook, and WhatsApp—37 questions)	31% reported weight gain. Participants’ dietary habits were not desirable (“unhealthy” dietary patterns). 36.2% declared spending five hours daily on screens (not for work). Unfortunately, 60.8% of the participants had sleep disturbances during the pandemic	[28]

**Table 3:** Brief summary of studies related to the consequences of the lockdown in European populations.

Population Studied	Sample Size (N)	Methodology of the Study	Impact/Changes Observed	Reference
Danish schoolchildren using a digital reading app	5485	Linking information from the Danish digital reading app BB to data on children’s sociodemographics	During initial lockdown, there was a general increase in reading activity, especially in children of college-educated parents and children of parents above the median income level, but long-term effects of lockdown were similar for all socioeconomic groups	[61]
Spanish schoolchildren and adolescents	860	Online survey answered by parents (Web-form structured questionnaire distributed using social media platforms)	Decrease in daily fruit and vegetable consumption and physical activity Increase in screen exposure and sleep time	[81]
Spanish schoolchildren and adolescents	240	Online survey (Validated structured questionnaire distributed to schools participating in the MUGI project)	Decrease in physical activity Increase in screen exposure and adherence to a Mediterranean diet	[82]
Irish adolescents	1214	Online survey (Self-reported questionnaire distributed to schools)	Decrease in physical activity	[83]
Italian children and adolescents with obesity	41	Twelve-question in-person and telephone interviews with parents of non-adults with obesity enrolled in the OBELIX Study	Increased intake of potato chips, red meat, sugary drinks, and fruit Similar vegetable consumption Decreased sports time Increased sleep and screen exposure	[84]
Italian adolescents	1568	Online survey (Online modified version of International Physical Activity Questionnaire (IPAQ))	Decrease in physical activity among inactive and moderately active adolescents Increased in physical activity on highly active adolescents	[85]
Italian adolescents	306	Online survey (Structured questionnaire in Google Forms)	Generally worse subjective psychological well-being Development of new interests and different routines and maintained social relationships	[24]
Italian adolescents	8	Clinical assessment and laboratory analysis	Development of primary chilblains	[86]
French university students	5738	Online survey (Structured questionnaire distributed to universities)	Increased binge eating in overweight students or students with obesity and in females, associated with high levels of stress Dietary restriction associated with stress and anxiety Aggravation of eating disorders	[25]
Bavarian university students (Germany)	1964	Online survey (Structured semi-quantitative questionnaire distributed to universities)	More confectionary and bread	[54]
Bavarian university students (Germany)	1980	Online survey (Structured semi-quantitative questionnaire distributed to universities)	Decreased physical activity and walking	[87]
Italian medicine students	1470	Online survey (Structured questionnaire distributed to student representatives)	Reduced total physical activity: more sedentary behavior, more sleep, and less walking Increased moderate and vigorous activity	[47]
Italian university students	1430	Online survey (Structured questionnaire proposed during academic lessons)	Reduced total physical activity, especially walking Increased sedentary behavior, especially use of electronic devices	[88]
Italian university students	176	Online survey (Structured questionnaire proposed during online academic lessons)	Positive association between physical activity and fruit, vegetables, and fish intake Association between cereal, legume, and low-fat meat consumption and both depression (positive association) and quality of life (negative association)	[59]
Italian university students	2125	Online survey (Structured questionnaire for the EPICO Study proposed during online academic lessons)	Reduced physical activity	[89]
Spanish university students	213	Online survey (Structured questionnaire distributed to universities)	Increased sedentary behavior and physical activity	[90]
Austrian apprentices and school students	1442 apprentices and 563 school students	Online survey (REDCap application was used)	Apprentices reported significantly more smoking than high school students and this difference was more pronounced in women. Alcohol consumption was higher in apprentices than school students, but only in women	[91]
United Kingdom new mothers	1365	Online survey (REDCap application was used. Links to the survey were being spread via websites, social media, and existing contacts)	Increased mental health problems	[92]
United Kingdom adults	2002	Online survey (Web-form structured semi-quantitative questionnaire)	Increased unhealthy eating in overweight individuals and individuals with obesity	[93]
United Kingdom adults	264	Online survey (Web-form structured questionnaire distributed via Facebook)	Increased problems in regulating eating and body image in patients with current or past eating disorders	[94]
Icelandic adults	97	Story completion method through a webpage	Amplification of tensions of parenthood, gender relations, paid work, housework, and childcare	[57]
Spanish adults	692	Telephone-based survey	Increased factors of risk for a depressive episode	[95]
Spanish adults	161	Online survey (Structured questionnaire in Google Forms after invitation via email)	Increased weight Decreased physical activity Worsening of sleep problems and self-perceived well-being	[11]
Spanish adults	3800	Online survey (Structured questionnaire after invitation via social media, email, and mobile phone)	Decreased physical activity Increased sedentary behavior	[46]
Spanish adults	1350	Online survey (Structured questionnaire in Microsoft Forms after invitation via WhatsApp app)	More fruits and vegetables, less processed food, and more eating and snacking	[96]
Spanish adults	1254	Online survey (Structured questionnaire after invitation via Facebook, WhatsApp, and Twitter)	Changes in lifestyle	[97]
Spanish adults	7514	Online survey (Structured questionnaire to participants of COVIDiet)	Better adherence to a Mediterranean diet	[98]
Spanish adults	1065	Online survey (Structured questionnaire in Google Forms after invitation via Facebook, WhatsApp, and Twitter)	Better adherence to a Mediterranean diet Increase in homemade desserts and pastries Decrease in physical activity	[99]
Spain	-	Analysis of public data	Higher environmental impact due to larger energy intake and lower nutritional quality of diets	[100]
Spanish adults	2741	Online survey (Structured questionnaire distributed via social media)	Increased and then decreased health risk behaviors	[27]
Spanish adults with pets	1297	Online survey (Structured questionnaire distributed via social media)	Emotional support by pets	[101]
Spanish adults	619	Online survey (Online anonymous survey system of Hospital Clinic of Barcelona)	Fewer coping strategies in patients with a psychiatric disorder	[26]
Spanish adults	3524	Online survey (Structured questionnaire distributed via social media)	Fewer coping strategies in younger adults	[14]
Croatian adults	3027	Online survey (Structured questionnaire distributed via social media)	Decreased physical activity and weight gain Fear, discouragement, and sadness	[22]
Italian adults	121	Outpatient clinic and phone interview	Increased weight gain Decreased physical activity Sleep problems	[15]
Italian adults	490	Online survey (Structured questionnaire in Google Forms distributed via social media)	Positive and negative changes in exercise, sleep, food, and tobacco consumption	[102]
Italian adults	2974	Online survey (Structured questionnaire in Google Forms distributed via social media)	Decreased physical activity Poorer psychological health and well-being	[17]
Italian adults	150	Online survey (Multiple-choice questionnaire distributed in the hospital)	Increased unhealthy eating in overweight individuals and individuals with obesity	[103]
Italian adults	3533	Online survey (EHLC-COVID19 questionnaire in Google Forms distributed in the hospital)	Weight gain Slightly increased physical activity Adherence to a Mediterranean diet	[104]
Italian adults	1932	Online survey (Structured questionnaire distributed via social media)	Increased eating, especially “comfort food”	[62]
Italian adults	602	Online survey (EHLC-COVID19 questionnaire in Google Forms distributed in the hospital)	Depressed mood, anxiety, hypochondria, and insomnia Consumption of “comfort food”	[18]
Italian adults and their children, with celiac disease	1983	Online survey (Structured questionnaire distributed to members of the Italian Celiac Society)	Maintained and improved adherence to gluten-free diet	[105]
Italian adults	268	Interview	Decreased physical activity	[106]
Polish adults	2618	Online survey (Structured questionnaire distributed via email and Facebook)	Fear of the pandemic, economic crisis, and worries about families	[20]
Polish adults	412	Online survey (Structured questionnaire distributed via social media)	Increased fears about the disease and decline in happiness and life satisfaction	[21]
Polish adults	2381	Online survey (Structured questionnaire in Google Forms distributed via social media)	Decreased physical activity Increased screen time and food consumption Changes towards prohealthy and unhealthy diets	[107]
Polish adults	312	Online survey (Structured questionnaire distributed via social media)	More snacking and more meals per day Changes in types of food consumed	[34]
Polish adults	1097	Online survey (Structured questionnaire distributed via social media)	More snacking and eating Changes in body weight gain Increased consumption of alcohol and tobacco	[108]
Polish women	1769	Online survey (Structured questionnaire distributed via social media)	Changes in body weight	[109]
Polish adults	1560	Online survey (Structured questionnaire distributed via a Polish online health service)	Increased physical activity in villages and towns below 5000 inhabitants Decreased physical activity in large cities	[110]
Irish Shed adult men	383	Survey completed with a research team member	Decline in happiness, life satisfaction, and physical activity Loneliness	[16]
Belgian adults	13,515	Online survey (Sstructured questionnaire distributed via email and social media)	Increased physical activity and sedentary behavior	[12]
Belgian adults	8640	Online survey (Structured questionnaire distributed via health-related websites and social media)	Food insecurity and bad dietary patterns	[111]
Dutch adults	1030	Online survey (Structured questionnaire distributed via email after recruitment by the panel agency)	Increased unhealthy eating in overweight individuals and individuals with obesity	[112]
French adults	11,391	Online survey (Structured questionnaire distributed via social media)	Increased alcohol, tobacco, and cannabis use, caloric/salty food intake, and screen exposure	[50]
French adults	1454	Online survey (Structured questionnaire distributed via social media)	More depressed, stressed, or irritable Increased alcohol and tobacco consumption More balanced diet	[113]
Lithuanian adults	2447	Online survey (Structured questionnaire to participants of COVIDiet)	More eating, snacking, and cooking at home Decreased physical activity Weight gain	[114]
Portuguese adults	1404	Online survey (Structured questionnaire in Google Forms distributed via social media and newspapers)	More anxiety in females and young adults	[115]
Scottish adults	399	Online survey (Structured questionnaire in Gorilla distributed via social media)	Increased bad mood	[116]
Adults from Bosnia and Herzegovina, Croatia, Greece, Kosovo, Italy, Serbia, Slovakia, Slovenia, and Spain	4108	Online survey (Structured questionnaire in 1KA distributed via social media)	More physical inactivity, sleep, screen exposure, and weight gain. More regular and healthier meals Decreased alcohol intake and smoking	[117]
Adults from Norway and Sweden	3508	Online survey (Validated structured questionnaire based in focus groups)	More food intake and sedentary behavior	[118]
Portuguese adults	43	Phone interview to Portuguese adults with eating disorders recruited in a hospital	Worsening of eating disorders	[119]
Dutch older adults	1119	Structured questionnaire paper, online or phone survey to participants of the LASA Study	Predisposition to overnutrition Decreased physical activity	[120]
French older adults	6	Semistructured qualitative interview with professionals	Reduced physical activity	[121]
Spanish older adults	483	Online survey (Structured questionnaire)	Regular exercise correlated with positive mood	[122]

**Table 4:** Brief summary of studies related to the consequences of the lockdown in North American populations.

Population Studied	Sample Size (N)	Methodology of the Study	Impact/Changes Observed	Reference
United States children	-	Analysis of missed meals and government programs	Risk of undernutrition since more than 1.15 billion meals were not served in school as a result of school closures during the 9-week period between 9 March and 1 May	[38]
United States children	8600	Survey (Geo-coded data on children and their families from the Early Childhood Longitudinal Study-Kindergarten Class of 2010–2011 (ECLS-K). ECLS families were asked questions)	Congruence between household and neighborhood education and race/ethnicity associates with the likelihood of experiencing food insecurity	[127]
United States university students	2039	Online survey (Emailed invitation and link to a 94-item online questionnaire through Qualtrics online survey software)	Changes in food security status experienced by students (12% improved, 68% stayed the same, and 20% worsened)	[128]
United States children	211	Online survey to parents and legal guardians (Invited through various social media platforms (e.g., Facebook, Twitter) and email list)	Decreased physical activity, mainly consisting on free play/unstructured activity (90% of children) and going for a walk (55% of children) Increased sedentary behavior (90 min of school-related sitting and over 8 h of leisure-related sitting a day)	[129]
Canadian children and adolescents	1472 answers	Online survey to parents (Maru/Matchbox consumer online database was used. Responses were reported using a 5-point Likert type scale)	Decreased physical activity Increased sedentary behavior and sleep (only 4.8% of children and 0.6% of youth were meeting combined movement behavior guidelines)	[130]
Canadian parents	361	Online survey (Completed questionnaires were received from 254 families)	Decreased physical activity, increased sedentary behavior, and more cooking at home and snacking among mothers, fathers, and children	[58]
United States adults	173	Online survey (Sent out via Facebook)	Weight gain and increased unhealthy dietary patterns	[131]
United States adults	17,234,452 observations	Data from 138,989 establishments	Increased unhealthy dietary patterns in adults with obesity	[132]
United States adults	3133	Online survey (71-item questionnaire conducted online through Qualtrics^XM^. Recruitment occurred through social media platforms and ResearchMatch)	Unchanged dietary patterns (43.6–87.4% of participants) Increased consumption of sweets (43.8%) and salty snacks (37.4%)	[133]
United States adults	3052	Online survey (Emails, snowball sampling and posts to social media pages were used)	Decreased physical activity (mean change: −32.3%) and increased screen exposure associated with bad physiological health	[134]
United States adult twins	3971	Online survey (Individuals from the Washington State Twin Registry)	Decreased physical activity (42% of participants) Increased anxiety in women	[135]
Canadian adults	4383	Online survey (The sample was a randomly selected subset of Labour Force Survey (LFS) respondents. A mailed a was prompting them to join)	Increased screen time (over 60% of participants) and alcohol (14%) and junk food (25%) consumption	[136]
Canadian adults	1098	Online survey (Snowball sampling using social media (Twitter, Facebook and LinkedIn) and regular media communications including stories in national and local media)	Differences between previously active and previously inactive individuals (40.5% of inactive individuals became less active, while 22.4% of active individuals became less active 33% of inactive individuals became more active, while 40.3% of active individuals became more active)	[137]
Canadian adults	1086	Online survey (Recruitment through regular media communications including stories in national and local media and snowball sampling using social media)	Decreased physical activity and increased anxiety in women	[13]

**Table 5:** Brief summary of studies related to the consequences of the lockdown in South American populations.

Population Studied	Sample Size (N)	Methodology of the Study	Impact/Changes Observed	Reference
Brazilian families with children aged less than 13 years	816 children	Online survey (Questionnaire based on LimeSurvey, free software to apply online questionnaires that can use databases for data persistence, housed in Faculdade de Motricidade Humana)	Reduction in the levels of physical activity among children. 38% reported that screen time was higher than in regular school hours, and 36.9% much higher 52.1% claimed to have more family activities than before isolation	[138]
Brazilian adults	45,161	Online survey (REDCap application was used)	Decrease in practicing physical activity Increase in screen time, intake of ultra-processed foods, smoking, and alcoholic beverage consumption	[49]
Brazilian adults	39,208	Online survey (REDCap application was used)	Participants with incidence of sedentary behaviors were more likely to follow an unhealthy diet	[139]
Brazilian adults (6881 with depression and 35,042 without depression)	41,923	Online survey ((A) Invitation through a chain sampling procedure (B) Information about the study was disseminated through press releases, social communications from participating research institutions, state health departments, and social media)	Participants with depression were more likely to present elevated frequency of ultra-processed food consumption	[140]
Chilean adults	700	Online survey (Survey was shared via institutional emails, Facebook, Instagram, WhatsApp, and Twitter in May and June 2020 (for eight weeks))	Sedentary time ≥ 6 h/day, low water consumption, and consumption of fried foods 3 times per week had a positive association with body weight increase. Middle socioeconomic background was associated with body weight increase Daily alcohol consumption was associated with physical activity decrease	[37]
Ecuadorian adults	1022	Online survey (Survey designed on the Google Forms platform (validated questionnaires))	Decreased physical activity, both in high and moderate intensity, while low intensity became predominant Mild improvement in eating habits	[141]
Ecuadorian adults	9522	Online survey ((A) Data were collected using Google Forms (B) The survey link was disseminated (i) on social networks such as Facebook, Instagram, and through WhatsApp (ii) through the official media of the Ecuadorian Universities: Universidad Estatal de Milagro (UNEMI) and the Escuela Superior Politécnica de Chimborazo (ESPOCH)).	Sleep quality differed according to sex (worse in women) Women had greater changes in the habitual consumption of food compared to men People 18–40 years of age decreased their food consumption in relation to people > 40 years	[142]

**Table 6:** Brief summary of studies related to the consequences of the lockdown in Oceanian populations.

Population Studied	Sample Size (N)	Methodology of the Study	Impact/Changes Observed	Reference
Australian students	509	Online survey (The Automated Self-Administered Dietary Assessment Tool (ASA24-Australia-2016) and the Active Australia Survey were used)	Females reported having greater energy intake. 30% fewer students reported following a lifestyle with sufficient levels of activity	[144]
Australian young people (aged 16–25)	371	Online survey (Participants were recruited via targeted advertisements on Facebook and Instagram posts from June to October 2020)	Participants reported high levels of psychological distress, with over 40% reporting severe levels of anxiety and depression. Young people also spent more time on social media as a result of the pandemic	[145]
New Zealanders adults	3028	Online survey ((A) Recruitment was through convenience/snowball sampling, promoted via social media (B) Stakeholders and colleagues from health and food-related organisations shared the survey invitation among their networks)	Decreased enjoyment of grocery shopping and increased home cooking as well as baking New unhealthy dietary patterns appeared	[48]

**Table 7:** Brief summary of studies related to the consequences of the lockdown in African populations.

Population Studied	Sample Size (N)	Methodology of the Study	Impact/Changes Observed	Reference
Kenyan and Ugandan adults	313 and 129	Online survey (The questionnaire was sent using social media (WhatsApp, Facebook, Telegram, and Twitter), and via email)	Evidence of worsening food security and dietary quality of respondents. Loss or reduction in income and reduced access to markets were observed	[41]
Low-income families in rural Kenya	328 low-income rural households	Weekly household interviews (Data were collected from all adults in the household, separately and in private. Diary data collection was preceded by a household survey, collecting baseline demographic, socio-economic, and health information)	Income from work decreased	[148]

**Table 8:** Brief summary of similar trends during the pandemic (i.e., the same effects observed in different countries).

Observed Effect in Many Countries	Continent	Population Studied	[Reference], Sample Size (N)
**Reduced physical activity**	Asia	Chinese students	[45], N = 10,416
		Chinese adults	[70], N = 2702
		Indian adults	[19], N = 995
		Thai adults	[64], N = 4460
**Reduced physical activity**	Europe	Spanish schoolchildren and adolescents	(a) [81], N = 860, (b) [82], N = 240
		Irish adolescents	[83], N = 1214
		Italian adolescents	[85], N = 1568
		Bavarian university students	[87], N = 1980
		Italian university students	(a) [47], N = 1470, (b) [88], N = 1430, (c) [89], N = 2125
		Spanish university students	[90], N = 213
		Spanish adults	(a) [46], N = 3800, (b) [11], N = 161, (c) [99], N = 1065
		Croatian adults	[22], N = 3027
		Italian adults	(a) [23], N = 121, (b) [17], N = 2974, (c) [106], N = 268
		Polish adults	[107], N = 2381
		Lithuanian adults	[114], N = 2447
		Dutch older adults	[120], N = 1119
**Reduced physical activity**	North America	United States children	[129], N = 211
		Canadian children and adolescents	[130], N = 1472
		Canadian adults	(a) [58], N = 361, (b) [13], N = 1086
		United States adults	[134], N = 3052
		United States adult twins	[135], N = 3971
**Reduced physical activity**	South America	Brazilian families with children aged less than 13 years	[138], N = 816 children
		Brazilian adults	[49], N = 45,161
		Ecuadorian adults	[141], N = 1022
**Weight gain**	Asia	Jordanian adults (and some students)	[35], N = 4388 (673 students)
		Chinese adults	[70], N = 2702
		Israel adults	[72], N = 1895
		Indian adults	[19], N = 995
		United Arab Emirates general population	[28], N = 1012
**Weight gain**	Europe	Croatian adults	[22], N = 3027
		Italian adults	(a) [23], N = 121, (b) [104], N = 3533
		Polish adults	[108], N = 1097
		Polish women	[109], N = 1769
		Lithuanian adults	[114], N = 2447
		Adults from Bosnia and Herzegovina, Croatia, Greece, Kosovo, Italy, Serbia, Slovakia, Slovenia, and Spain	[117], N = 4108
**Weight gain**	North America	United States adults	[131], N = 173
**Weight gain**	South America	Chilean adults	[37], N = 700
**Increase in screen exposure**	Asia	Chinese students	[45], N = 10,416
		Jordanian children and adolescents	[69], N = 477
		Indian adults	[19], N = 995
		United Arab Emirates	[28], N = 1012
**Increase in screen exposure**	Europe	Spanish schoolchildren and adolescents	(a) [81], N = 860, (b) [82], N = 240
		Italian children and adolescents with obesity	[84], N = 41
		Italian university students	[88], N = 1430
		Polish adults	[107], N = 2381
		French adults	[50], N = 11,391
		Adults from Bosnia and Herzegovina, Croatia, Greece, Kosovo, Italy, Serbia, Slovakia, Slovenia, and Spain	[117], N = 4108
**Increase in screen exposure**	North America	United States adults	[134], N = 3052
		Canadian adults	[136], N = 4383
**Increase in screen exposure**	South America	Brazilian families with children aged less than 13 years	[138], N = 816 children
		Brazilian adults	[49], N = 45,161

## Data Availability

Not applicable.
